# Assessment of density functional approximations for N_2_ and CO_2_ physisorption on benzene and graphene

**DOI:** 10.1002/jcc.26945

**Published:** 2022-06-06

**Authors:** Víctor M. Rayón, Iván Cabria

**Affiliations:** ^1^ Departamento de Química Física y Química Inorgánica, Facultad de Ciencias Universidad de Valladolid Valladolid Spain; ^2^ Departamento de Física Teórica, Atómica y Óptica, Facultad de Ciencias Universidad de Valladolid Valladolid Spain

**Keywords:** DFT, dispersion interactions, gas storage, graphene, physisorption, pore size distribution

## Abstract

Experimental isotherms of N_2_ and CO_2_ on carbon‐based porous materials and models of the physisorption of gases on surfaces are used to obtain the pore size distribution (PSD). An accurate modelization of the physisorption of N_2_ and CO_2_ on the surface of carbon‐based porous materials is important to obtain accurate N_2_ and CO_2_ storage capacities and reliable PSDs. Physisorption depends on the dispersion interactions. High precision ab initio methods, such as CCSD(T), consider accurately the dispersion interactions, but they are computationally expensive. Double hybrid, hybrid and DFT‐based methods are much less expensive. In the case of graphene, there are experimental data of the adsorption of N_2_ and CO_2_ on graphite that can be used to build the Steele interaction potential of these gases on graphene. The goal is to find out hybrid and/or DFT methods that are as accurate as the CCSD(T) on benzene and as accurate as the experimental results on graphene. Calculations of the interaction energy curves of N_2_ and CO_2_ on benzene and graphene have been carried out using the CCSD(T) method and several double hybrid, hybrid, and DFT methods that consider the dispersion interactions. The energy curves on benzene have been compared to the CCSD(T) and the energy curves on graphene have been compared with the Steele energy curves. The comparisons indicate that double hybrids with dispersion corrections and *ω*B97 based DFT methods are accurate enough for benzene. For graphene, only the PBE‐XDM functional has a good agreement with the Steele energy curves.

## Graphical abstract

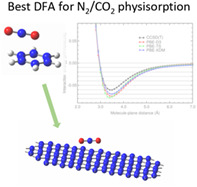

## 1 INTRODUCTION

N_2_ and CO_2_ gases are used to characterize solid porous materials like, for instance, activated carbons, graphite fibers, and so forth. Obtaining the pore size distributions (PSDs), of these materials is an important part of the characterization. An accurate PSD is relevant for optimal gas storage and separation applications. Unfortunately, this is a complicated task due to the complex structure of these materials and to the indirect methods used to obtain the PSD.

Most of the indirect methods consist on using experimental and theoretical isotherms of a gas inside slit‐shaped pores of different widths, to solve an adsorption integral equation. The PSD is the solution of the equation. The indirect methods of this type differ on (a) the procedure to obtain the theoretical isotherms and/or (b) the procedure and details to solve the adsorption integral equation. The first method of this type was proposed by Seaton et al.^1^ and they used a local functional to obtain the isotherms. This method was improved by the Non‐Local Density Functional Theory, NLDFT, method,^2^, ^3^, ^4^, ^5^ based on hard spheres and a non‐local density functional. The Quenched Solid Density Functional Theory, QSDFT, method^6^ and variations or new generations of the NLDFT method^7^, ^8^ were developed to eliminate the artifacts (gaps and peaks) of the PSDs obtained using the NLDFT method. The NLDFT methods are the most used methods to obtain the PSD.

Some indirect methods of this type^9^, ^10^, ^11^ obtain the theoretical isotherms of the slit‐shaped pores from Grand Canonical Monte Carlo, GCMC, simulations, using Lennard‐Jones, LJ,^12^ and/or Steele potentials^13^ and then, they solve the adsorption integral equation. These are called GCMC methods. The LJ potential is used to simulate the interactions between the molecules and the interactions of the molecules with the atoms of the porous material. The Steele potential is used to simulate the interaction between the molecules and a graphene layer or a slab of graphene layers. Samios et al.^14^ proposed a method that does not use the adsorption integral equation. The method uses GCMC simulations made with LJ potentials^12^ to obtain the theoretical isotherms of slit‐shaped pores of different widths and an initial postulated PSD, which is changed iteratively until an agreement with the experimental isotherm of the porous material is reached.

The PSD obtained by means of any indirect method depends on the interaction potential or on the density functional used to obtain the isotherms. An interaction potential more accurate than the LJ potentials and than the non‐local functional based on hard spheres of the NLDFT model could yield more accurate (a) theoretical isotherms and (b) PSDs of solid nanoporous materials.

The goal of this paper is to find accurate DFT or hybrid methods that can be used to calculate the 3d‐interaction potential energy of N_2_ and CO_2_ on carbon‐based solid porous materials. More specifically, the goal is to find the DFT or hybrid method that yields the most accurate interaction energy curves of N_2_ and CO_2_ on benzene, compared with the CCSD(T) energy curves, and on graphene, compared with the Steele potential energy curves of N_2_ and CO_2_ on graphene, obtained from experimental data. The most accurate DFT and hybrid methods will be used to run first principles calculations of the interaction of N_2_ and CO_2_ on carbon‐based nanoporous materials, saving memory and computer time. The 3d interaction potential energy obtained in those calculations will be used to run GCMC simulations of the N_2_ and/or CO_2_ isotherms of the carbon‐based nanoporous materials. These accurate theoretical isotherms, in turn, will be used to obtain more accurate PSDs.

Graphene and benzene have been selected as carbon‐based surfaces, because they have well geometrically defined surfaces and because slit‐shaped pores are made of graphene layers. The Steele interaction energy curve^13^ of N_2_ and CO_2_ on graphene is, due to its experimental origin and to experimental data available, the best option to study accurately the interactions of these molecules on graphene or in slit‐shaped pores based on graphene layers. However, for realistic carbon‐based solid porous materials, the Steele potential cannot be used. The Coupled Cluster Single and Double (Perturbative Triple), CCSD(T), method takes into account accurately the weak interactions of molecules, H_2_, N_2_, CO_2_, and so forth, on a surface, while the density functional theory, DFT, methods do not include those interactions or include them with different degrees of complexity. Hybrid and double‐hybrid DFT functionals include with some degree of accuracy the weak interactions. The computational cost of the CCSD(T), double‐hybrid, hybrid, and DFT methods scales as O(*N* ^7^), O(*N* ^5^), O(*N* ^4^), and O(*N* ^3^), respectively, where *N* is proportional to the system size. The amount of atoms of many carbon‐based solid porous materials makes unfeasible to run CCSD(T) calculations, while DFT and hybrid calculations of the interaction of a molecule with those materials are feasible.

In a former publication,^15^ we analyzed the energy curves of H_2_ on benzene, obtained with the CCSD(T) method and several DFT methods. The present research is, somehow, a continuation of the H_2_ on benzene research. Of particular relevance to this study is a previous work by Witte and co‐workers^16^ in which several density functional approximations (DFAs) have been assessed against the results of benchmark CCSD(T) calculations using the CO_2_‐benzene complex. The present study assesses a larger set (28 vs. 15) of DFAs, including double‐hybrids, the interaction of N_2_ in addition to the interaction of CO_2_, and extends the analysis to the physisorption of both moieties on graphene. Up to our knowledge, no assessment of such a large set of functionals has been performed so far on these systems.

The structure of this paper is as follows. The methodology used in this study is described in Section 2, along with the computational details. The computational results are presented and discussed in Section 3. This includes the assessment of DFAs on benzene‐CO_2_, benzene‐N_2_, graphene‐CO_2_, and graphene‐N_2_. Finally, Section 4 contains a summary and some conclusions of this study.

## 2 METHODOLOGY

### 2.1 Configurations of the molecules on the substrates

**FIGURE 1:**
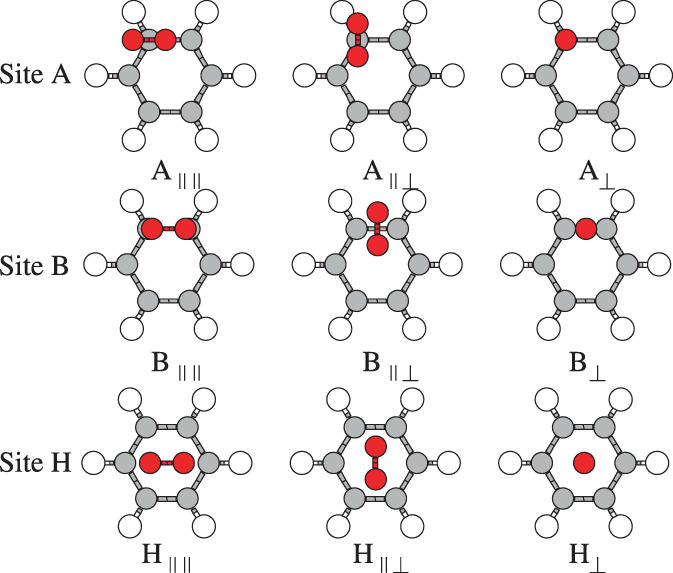
The nine configurations of the N_2_ molecule on top of the benzene molecule. Three sites: on carbon atom, A, on carbon–carbon bond, B, and on the center of the hexagon, H. For each site, three different orientations of the molecular axis of N_2_ are explored

**FIGURE 2:**
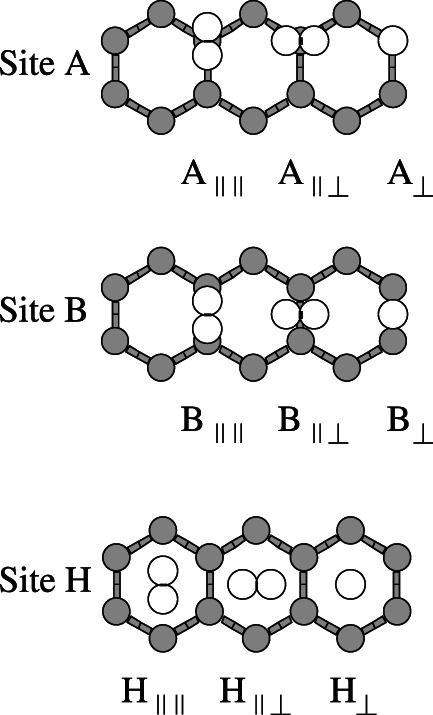
The nine configurations of the N_2_ molecule on top of the graphene surface. Three sites: on carbon atom, A, on carbon–carbon bond, B, and on the center of the hexagon, H. For each site, three different orientations of the molecular axis of N_2_ are explored

The molecules N_2_ and CO_2_ were placed on top of the three main sites of the substrates (benzene or graphene): The center of an hexagon, H, the center of a C—C bond, B, and a carbon atom, A. There are three main relative orientations of the molecular axis of the molecule: The axis perpendicular to the substrate plane, ⊥, the axis parallel to the substrate plane and parallel to two C—C bonds of the hexagon, ∥∥, and the axis parallel to the substrate plane and perpendicular to two C—C bonds of the hexagon, ∥⊥. The combination of site and orientation is a configuration. There are nine main configurations. The following notation has been used for the configurations: The letter indicates the site where the molecule was placed and the set of symbols after the letter, denotes the relative orientation of the molecular axis. An additional possible configuration in which the main axis of N_2_ or CO_2_ orients itself parallel to the benzene surface on top of a C—H bond has not been considered since this particular configuration is not relevant for graphene. The nine configurations considered are depicted in Figures 1 and 2 for N_2_ on benzene and graphene, respectively, but they are also valid for CO_2_.

### 2.2 General procedures applied to all the computational methods

Four codes have been used to carry out the present calculations of N_2_ and CO_2_ on benzene and graphene: Gaussian 16 (G16RevA.03), Orca 4.2.1, Molpro 2018.2, and Quantum Espresso (QE) 6.3. DFT and DFT‐based methods that include dispersion interactions are implemented in these codes. The same procedures have been used with the four codes: For each of the methods used, the geometries of N_2_, CO_2_, benzene, and graphene were optimized. The geometry optimization was performed until the forces acting on the atoms were less than the threshold values depending on the code. We provide the specific values in the corresponding subsections in the Supporting Information.

After the optimization of the geometries of N_2_, CO_2_, benzene, and graphene for each method, the most stable configuration of each molecule, N_2_ or CO_2_, on a substrate, benzene or graphene, was searched for, using one of the first principles method for all the configurations depicted in Figures 1 and 2. Four systems (a system is a molecule on a substrate) have been studied and for each system, the most stable configuration was obtained. Then, the interaction energy curve for each method and system was calculated, with the molecule on the most stable configuration of the system, obtained in the last step.

The interaction energy *E*(*d*) between benzene or graphene and a N_2_ or CO_2_ molecule placed at a distance *d* from the benzene or graphene plane (*d* is the distance between the center of mass of the molecule and the benzene or graphene plane) is defined, for all the computational codes, in terms of the energies of the interacting system and the separated components as:

(1) $$E\left(d\right)=E\left(\text{molecule}\;\mathrm{on}\;A;d\right)-E\left(\text{molecule}\right)-E\left(A\right),$$

where, molecule can be N_2_ or CO_2_ and *A* can be benzene or graphene. The binding energy *E*_*b*_ is the interaction energy at the equilibrium molecule‐benzene or molecule‐graphene plane distance *d*_*e*_, that is, *E*_*b*_ = *E*(*d*_*e*_). A negative value of *E*_*b*_ means that the molecule is bonded to benzene or to graphene. The interaction energy curve is the set of pairs or points (*d*, *E*(*d*)).

To obtain the most stable configuration and also to obtain the interaction energy curve, the individual geometries of benzene or graphene, N_2_ and CO_2_ were kept frozen, with the optimized geometries obtained in the optimization process for the corresponding method, and only the distance between the center of mass of N_2_ or CO_2_ and the benzene or graphene plane was changed. That distance was changed around the interval 3–4 Å to obtain the most stable configuration and, to obtain the interaction energy curve, that distance was changed from 2 to 7 Å, with a step of 0.1 Å.

Table 1 lists the 28 DFAs tested in the present work along with the computational codes used for the calculations. The set of DFAs comprise 18 GGAs, 6 hybrids, and 3 double hybrids. Besides, the local VWN5 was also tested.

**TABLE 1:** DFAs tested in the present study (see the text for further details on the DFAs and the computational codes)

DFA	Rung	Program	DFA	Rung	Program
VWN5	LDA	Gaussian	*ω*B97M‐D3BJ	Hybrid	Orca
VWN‐RPA	LDA	QE	*ω*B97M‐V	Hybrid	Orca
PW91	GGA	Gaussian	B2PLYP‐D3	Double hybrid	Orca
PBE	GGA	Gaussian	DSD‐BLYP	Double hybrid	Orca
rev‐PBE	GGA	QE	DSD‐BLYP‐D3BJ	Double hybrid	Orca
B97D	GGA	Gaussian	vdW‐DF	GGA	QE
B97‐D3	GGA	Gaussian	vdW‐DF‐C09	GGA	QE
PBE‐D2	GGA	Gaussian	vdW‐DF‐cx	GGA	QE
PBE‐D3	GGA	QE	optB86b‐vdW	GGA	QE
PBE‐TS	GGA	QE	optB88‐vdW	GGA	QE
PBE‐XDM	GGA	QE	vdW‐DF2	GGA	QE
MN15	Hybrid	Gaussian	rev‐vdW‐DF2	GGA	QE
*ω*B97X‐D	Hybrid	Gaussian	vdW‐DF2‐C09	GGA	QE
*ω*B97X‐D3BJ	Hybrid	Orca	RVV10	GGA	QE
*ω*B97X‐V	Hybrid	Orca			

The use of atom‐centered finite basis sets in DFT calculations in Gaussian, Orca, and Molpro gives rise to the well known Basis Set Superposition Error (BSSE). For this reason, the interaction energies have been calculated always using the counterpoise (CP) correction method of Boys and Bernardi.^17^ On the other hand, Symmetry Adapted Perturbation Theory computes the interaction (binding) energy directly (no substractions whatsoever) and is therefore free from BSSE. Likewise, plane‐waves based calculations (those performed using Quantum Espresso in this study) are inherently also free of BSSE.

The atom–centered all–electron aug‐cc‐pVTZ basis set^18^, ^19^, ^20^, ^21^, ^22^ was used for this study. This is an augmented (i.e., it contains diffuse functions) triple–zeta basis which is well suited for the study of these systems. We have nevertheless assessed the quality of this basis by comparing its performance to the larger aug‐cc‐pVQZ basis set. The result, collected in the Supporting Information, shows that both sets provide very similar results (the difference between the aug‐cc‐pVTZ and aug‐cc‐pVQZ interaction energies is smaller than 10^−3^ eV/molecule in the region near the minimum, and it becomes negligible at larger N_2_‐benzene distances).

A short description of the DFAs tested in this study along with technical details on the calculations performed with the four codes used in this work are provided as Supporting Information.

### 2.3 Calculation of the errors of the interaction energy curves of N_2_ and CO_2_ on benzene and graphene

In a previous publication we used a quantitative methodology to compare the interaction energy curves of H_2_ on benzene and graphene.^15^ We have used the same methodology in the present research to obtain a numerical or quantitative comparison of the interaction energy curves of N_2_ and CO_2_ on benzene. In that methodology, the amounts RMSE_*x*_, the root‐mean‐square error, and RMSPE_*x*_, the root‐mean‐square percentage error, (*x* = *m* or *x* = *t*) for the method *F* are defined as:

(2) $${\text{RMSE}}_{x}=\sqrt{\sum_{i_{x}=1}^{N_{x}}\frac{{\left(E{\left(d_{i_{x}}\right)}_{F}-E{\left(d_{i_{x}}\right)}_{\text{CCSD}\left(T\right)}\right)}^{2}}{N_{x}}},$$

(3) $${\text{RMSPE}}_{x}=100\sqrt{\sum_{i_{x}=1}^{N_{x}}\frac{{\left(E{\left(d_{i_{x}}\right)}_{F}-E{\left(d_{i_{x}}\right)}_{\text{CCSD}\left(T\right)}\right)}^{2}}{{\left(E{\left(d_{i_{x}}\right)}_{\text{CCSD}\left(T\right)}\right)}^{2}N_{x}}},$$

where, *x* = *m* stands for the region around the minimum of the interaction energy and *x* = *t* stands for the tail region. The ranges of the molecule‐plane distances $d_{i_{x}}$ used to calculate RMSE and RMSPE of the interaction energies of N_2_ and CO_2_ on benzene are shown in Table 2.

**TABLE 2:** Intervals of molecule‐plane distances (in Å) used to calculate the errors RMSE and RMSPE in Equations (2) and (3) of the interaction energy curves of N_2_ and CO_2_ on benzene and graphene

Substrate	Molecule	Range of $d_{i_{m}}$	Range of $d_{i_{t}}$
Benzene	N_2_	3.1–4.2	4.3–7.0
Benzene	CO_2_	2.9–4.2	4.3–7.0
Graphene	N_2_	3.0–4.3	4.4–7.0
Graphene	CO_2_	2.9–4.0	4.1–7.0

In the case of graphene, we have used as reference, the Steele potential energy curve of N_2_ and CO_2_ on graphene,^13^ instead of the CCSD(T) energy curve in Equations (2) and (3). The shape of the Steele potential is based on experimental observations. The values of the parameters of the C‐N_2_ and C‐CO_2_ interactions used in the Steele potentials of N_2_ and CO_2_ on graphene, were obtained from experimental data of N_2_ and CO_2_ on *graphite* as follows.

Vidali et al.^23^ published a review of the binding energies and equilibrium distances of several molecules on different surfaces, including the surface of graphite, obtained from many experiments. The binding energy and equilibrium distance of N_2_ on *graphite* are −0.104 ± 0.003 eV and 3.34 Å, respectively, and the binding energy and equilibrium distance of CO_2_ on *graphite* are −0.1784 eV and 3.2 ± 0.1 Å, respectively, according to the experiments reviewed by Vidali et al.^23^ The Steele potential can be the potential of a molecule on a slab made of a finite or an infinite number of graphene layers.^13^ This potential depends on the parameters *ϵ* and *σ* of the C‐molecule interaction.

We ran calculations of the Steele potential of N_2_ and CO_2_ on graphite, simulating the graphite as a slab made of 101 graphene layers, separated the experimental distance between layers on graphite, 3.35 Å, for different values of the parameters *ϵ* and *σ* of the C‐N_2_ and C‐CO_2_ interaction, searching for the values that yielded a Steele potential of N_2_ on graphite with a binding energy and an equilibrium distance that matched their respective experimental values. The obtained values for C‐N_2_ were *ϵ* = 65.3*k*_*B*_ and *σ* = 3.36 Å. The parameters obtained in the present work were used later to calculate the Steele potential energy curve of N_2_ on graphene, a single layer. The minimum of the Steele potential energy curve of N_2_ on graphene is at 3.4 Å and −0.0912 eV/mol.

The values of the parameters of the C‐CO_2_ interaction used in the Steele potential of CO_2_ on graphene, were obtained using the same procedure as in the case of N_2_. The values of the parameters of the C‐CO_2_ interaction of the Steele potential obtained in the procedure were *ϵ* = 123.2*k*_*B*_ and *σ* = 3.22 Å, as mentioned before. The equilibrium CO_2_‐graphene distance and binding energy of the Steele energy curve of CO_2_ on graphene are 3.2 Å and − 0.1584 eV/mol, respectively.

In Table 3 we compare the *ϵ* and *σ* parameters for the Steele potential of N_2_ and CO_2_ on graphite obtained in the present work, with those published in the scientific literature. We compare also the equilibrium distance and minimum of the Steele potential corresponding to those parameters with the experimental values of the interaction of N_2_ and CO_2_ on graphite. The C‐N_2_ and C‐CO_2_ parameters are obtained using the Lorentz‐Berthelot rules.^28^, ^29^ In one instance,^26^ only the C‐N_2_ and C‐CO_2_ parameters are reported. The parameters for N_2_ are similar to the obtained with present procedure. There is some disagreement with the parameters for CO_2_. However, the present obtained parameters agree better with the experiments of N_2_ and CO_2_ on graphite published by Vidali et al.^23^

**TABLE 3:** Parameters *ϵ* (in k_B_ units) and *σ* (in Å) of C, N_2_, CO_2_, C‐N_2_, and C‐CO_2_, and equilibrium distance *d*_*e*_ (in Å) and minimum *E*(*d*_*e*_) (in eV) of the Steele potential energy curve of N_2_ and CO_2_ on graphite corresponding to those parameters

*ϵ*_C_	*σ*_C_	$\epsilon_{N_{2}}$	$\sigma_{N_{2}}$	$\epsilon_{C-N_{2}}$	$\sigma_{C-N_{2}}$	*d*_*e*_	*E*(*d*_*e*_)	Ref.
43.45	3.4^27^	96.92	3.66^28^	64.89	3.53	3.51	−0.116	24, 25
–	–	–	–	63.15	3.36	3.34	−0.101	26
	–	–	–	65.3	3.36	3.34	−0.104	Present work
	–	–	–	–	–	3.34	−0.104	Experiments^23^

*ϵ*_C_	*σ*_C_	$\epsilon_{{\mathrm{CO}}_{2}}$	$\sigma_{{\mathrm{CO}}_{2}}$	$\epsilon_{C-{\mathrm{CO}}_{2}}$	$\sigma_{C-{\mathrm{CO}}_{2}}$	*d*_*e*_	*E*(*d*_*e*_)	Ref.
43.45	3.4^27^	230.56	3.83^28^	100.09	3.62	3.59	−0.1892	24, 25
43.45	3.4^27^	236.1	3.75^29^	101.28	3.58	3.56	−0.1866	24, 27
–	–	–	–	123.2	3.22	3.2	−0.1784	Present work
–	–	–	–	–		3.2	−0.1784	Experiments^23^

## 3 RESULTS

### 3.1 Optimization of the geometries of N_2_ , CO_2_ , benzene and graphene

We have optimized the geometries of N_2_, CO_2_, benzene and graphene using CCSD(T) and the DFAs collected in Table 1. The optimized geometries are compared to the experimental data and the performance of the different DFAs are discussed in the Supporting Information. Let us just highlight here that all the tested DFAs describe correctly the equilibrium geometries being most of them within 0.0060 Å from the experimental values. We finally remind the reader that the optimized geometries of the monomers for each method (CCSD(T) of DFA) were kept frozen in the calculation of the interaction energy of N_2_ and CO_2_ on benzene and graphene.

### 3.2 Calculations of N_2_ physisorbed on benzene: DFT‐based and hybrid methods versus CCSD(T) method

The first step of the calculations of N_2_ on benzene consisted on finding the most stable or optimal configuration of the N_2_ molecule on benzene. The equilibrium and binding energies for the nine configurations of N_2_ on benzene obtained in CCSD(T) calculations are reported in Table 4.

**TABLE 4:** Equilibrium N_2_‐benzene plane distances, *d*_*e*_, in Å, and binding energies, *E*_*b*_, in eV, for N_2_ on different sites on benzene and with different orientations of the molecular axis, obtained with the CCSD(T) method, the counter poise method and the aug‐cc‐pVTZ basis set

Configuration	*d*_*e*_	*E*_*b*_
A_∥∥_	3.6	−0.0407
A_∥⊥_	3.6	−0.0404
A_⊥_	4.2	−0.0162
B_∥∥_	3.6	−0.0438
B_∥⊥_	3.6	−0.0437
B_⊥_	4.2	−0.0173
H_∥∥_	3.45	−0.0608
H_∥⊥_	3.45	−0.0608
H_⊥_	3.9	−0.0311

CCSD(T) calculations of N_2_ on benzene in the configuration H_⊥_ using the aug‐cc‐pVTZ basis set and the counterpoise method, yielded a binding energy of −0.0311 eV at an equilibrium molecule‐plane distance of 3.9 Å. The binding energies of the configuration H_∥∥_ and H_∥⊥_ are −0.0608 at the same equilibrium molecule‐plane distance, 3.45 Å, according to CCSD(T) calculations, using the counterpoise method and the aug‐cc‐pVTZ basis set. The difference is very small, 2.72 × 10^−5^ eV, and identical to the energy converge tolerance, 2.72 × 10^−5^ eV. We have selected the configuration H_∥∥_ is the most stable configuration and all the DFT and hybrid calculations of N_2_ on benzene have been carried out on this configuration.

A previous study on this complex by Jaeger et al.^30^ using also a CCSD(T)/aug‐cc‐pVTZ level of theory, although different geometries of the monomers, found a binding energy of 0.0606 eV and an equilibrium distance of 3.44 Å for the H_∥∥_ and H_∥⊥_ configurations.

The second step on this research consisted on calculations to obtain the equilibrium distance and binding energy with DFT and hybrid methods, with the N_2_ molecule on the most stable configuration on benzene, H_∥∥_. The N_2_‐benzene plane equilibrium distances and the binding energies obtained are given in Table 5.

**TABLE 5:** Equilibrium N_2_‐benzene plane distances, *d*_*e*_, in Å, and binding energies, *E*_*b*_, in eV, for N_2_ parallel to the benzene molecule, obtained with different theoretical methods. RMSE_*m*_ and RMSE_*t*_ are in eV, and RMSPE_*m*_ and RMSPE_*t*_ are in % (see text for their meaning)

Method	*d*_*e*_	*E*_*b*_	RMSE_*m*_	RMSPE_*m*_	RMSE_*t*_	RMSPE_*t*_
CCSD(T)	3.4	−0.0608	0.0000	0	0.0000	0
DFSAPT	3.5	−0.0587	0.0021	5	0.0005	5
VWN5	3.1	−0.1032	0.0287	68	0.0053	54
PW91	3.8	−0.0296	0.0346	74	0.0026	37
PBE	3.8	−0.0189	0.0444	94	0.0074	58
revPBE	4.5	−0.0067	0.0898	193	0.0096	60
B97D	3.5	−0.0538	0.0054	11	0.0029	27
B97‐D3	3.4	−0.0732	0.0123	26	0.0049	35
PBE‐D2	3.3	−0.0697	0.0098	23	0.0004	6
PBE‐D3	3.5	−0.0749	0.0133	27	0.0038	20
PBE‐TS	3.4	−0.0782	0.0134	27	0.0024	23
PBE‐XDM	3.5	−0.0698	0.0088	18	0.0039	47
MN15	3.4	−0.0811	0.0159	33	0.0056	62
*ω*B97X‐D	3.4	−0.0596	0.0021	5	0.0006	5
*ω*B97X‐D3BJ	3.4	−0.0742	0.0111	24	0.0007	4
*ω*B97X‐V	3.4	−0.0707	0.0082	17	0.0013	6
*ω*B97M‐D3BJ	3.4	−0.0683	0.0070	16	0.0006	7
*ω*B97M‐V	3.4	−0.0690	0.0075	16	0.0012	9
B2PLYP‐D3	3.4	−0.0667	0.0054	12	0.0005	6
DSD‐BLYP	3.4	−0.0536	0.0073	15	0.0032	22
DSD‐BLYP‐D3BJ	3.4	−0.0673	0.0065	15	0.0004	5
vdW‐DF	3.6	−0.0823	0.0226	50	0.0127	93
vdW‐DF‐C09	3.4	−0.0759	0.0170	38	0.0098	88
vdW‐DF‐cx	3.6	−0.0758	0.0184	41	0.0114	89
optB86b‐vdW	3.4	−0.0807	0.0203	44	0.0092	79
optB88‐vdW	3.4	−0.0790	0.0174	37	0.0065	61
vdW‐DF2	3.5	−0.0759	0.0127	25	0.0026	18
rev‐vdW‐DF2	3.5	−0.0626	0.0028	6	0.0012	6
vdW‐DF2‐C09	3.7	−0.0334	0.0262	54	0.0014	8
RVV10	3.4	−0.0729	0.0110	24	0.0015	24

**FIGURE 3:**
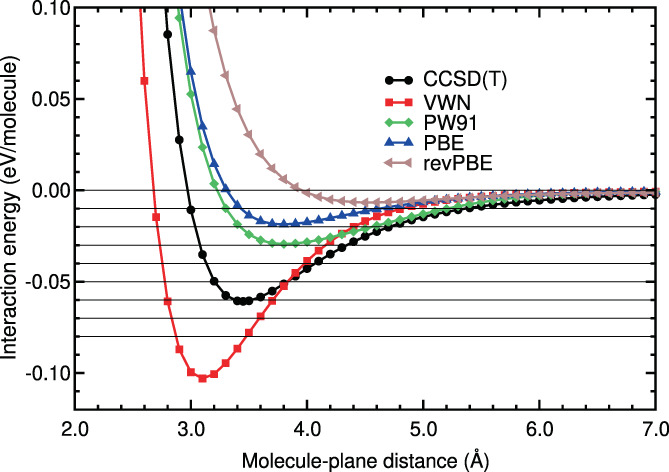
Interaction energy between N_2_ and benzene as a function of the N_2_‐benzene plane distance, obtained in CCSD(T), LDA, and GGA calculations

**FIGURE 4:**
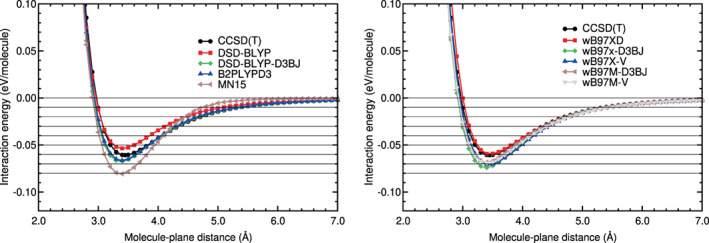
Interaction energy between N_2_ and benzene as a function of the N_2_‐benzene plane distance, obtained in CCSD(T) and hybrid calculations

**FIGURE 5:**
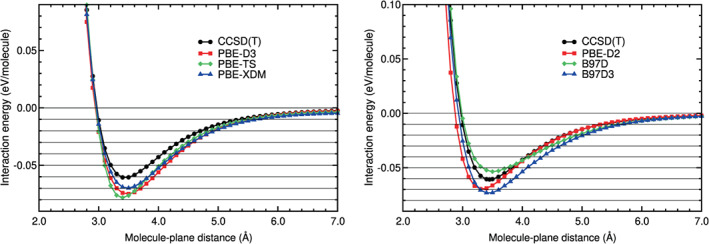
Interaction energy between N_2_ and benzene as a function of the N_2_‐benzene plane distance, obtained in CCSD(T), DFT‐D, and DFT‐D (G16) calculations

**FIGURE 6:**
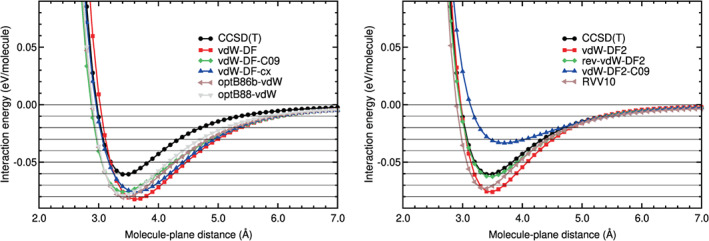
Interaction energy between N_2_ and benzene as a function of the N_2_‐benzene plane distance, obtained in CCSD(T), vdW‐DF, vdW‐DF2, and RVV10 calculations

The last step consisted on obtaining not only the location and value of the binding energy, but also the whole interaction energy curve. The interaction energy is defined in Equation (1), with molecule = N_2_ and *A* = benzene. The interaction energy curves of N_2_ on benzene obtained in the calculations are plotted in Figures 3, 4, 5, 6. The N_2_ molecule was in the H_∥∥_ configuration. Some horizontal lines corresponding to 0, −0.01, −0.02, −0.03, −0.04 eV, and so forth have been plotted in those figures, to guide the eye in the comparison of the different curves.

The values of the RMSE and RMSPE quantities, defined in Equations (2) and (3), have been also calculated for benzene–N_2_ and collected in Table 5. In the following lines, we compare the performance of the different DFAs we have assessed with respect to the CCSD(T) reference values.

Figure 3 and Table 5 show that VWN5 overestimates the CCSD(T) binding energy and the energies of the binding region, while GGA functionals (PW91, PBE and revPBE) underestimate the binding energy and the energies of the binding region as it was found on many other systems with weak interactions. The smallest RMSE in the binding region is 0.0287 eV (VWN5) which represents 68% RMSPE. In the tail region (beyond approximately 4–5 Å), a region which is particularly important for simulations of the N_2_ storage and adsorption on large carbon pores, the best performer is PW91, but still with a large RMSE of 0.0026 eV (37%). Clearly, LDA and GGA interaction energy curves of N_2_ on benzene are very different from the CCSD(T) curve (see Figure 3). This result was expected, since these functionals do not include the dispersion interactions, responsible of the physisorption interaction energy curves of N_2_ on benzene.

The interaction energy curves of the hybrid methods are, in general, much more similar to the CCSD(T) energy curve than the curves obtained with the other methods, as can be noticed in Figure 4 and in Table 5. The RMSEs of the hybrid methods around the equilibrium distance are well below 0.01 eV and RMSPEs equal or below 17%, except for *ω*B97X‐D3BJ and MN15, whose RMSEs and RMSPEs are above those values. In the long tail, the agreement with the reference is particularly very good, except for DSD‐BLYP, which does not include explicit dispersion corrections, and, again, MN15. In this region, the RMSE is below 0.0013 eV for the best performers, which represents RMSPE below 10%.

The DFT‐D or semiempirical method curves (see Figure 5) show similar deviations in the binding and tail regions. The best performers in the binding region are B97D and PBE‐XDM with RMSE below 0.01 eV and RMSPE below 20%. In the long tail, however, these DFAs perform poorly (RMSE 0.0029 and 0.0039 eV, which represents RMSPE above 27%). The best DFA in the long tail is PBE‐D2, with RMSE 0.0004 eV and RMSPE 6%.

The vdW‐DF methods include the dispersion interactions through non‐local functionals and are more complex than the semiempirical methods. However, the vdW‐DF curves do not coincide with the CCSD(T) curve in both regions: the binding and the tail region. The vdW‐DF curves are very different from the CCSD(T) curve (see Figure 6). In the binding region the RMSE of the vdW‐DF methods lies between 0.017 and 0.023 eV and the RMSPE between 37% and 50%. The behavior in the long tail region is even worse, with RMSEs larger than 0.006 eV, which represents RMSPEs above 60%.

As regards to the vdW‐DF2 family of methods, which also include the dispersion interactions through non‐local functionals, the energy curves agree well with the CCSD(T) energy curve in the tail region: The RMSEs of this group of methods are between 0.0012 and 0.0026 eV and the RMSPEs between 6% and 24%. In the binding region, however, there is not a general trend of this family of methods. The rev‐vdW‐DF2 curve coincides very well with the CCSD(T) curve (RMSE of 0.0028 eV and RMSPE of 6%), the vdW‐DF2‐C09 curve has high values of RMSE and RMSPE (0.0262 eV and 54%, respectively) and the vdW‐DF2 and RVV10 have a regular performance at the binding region, with RMSEs and RMSPEs around 0.011% and 24%, respectively. Finally, the DFSAPT curve is very similar to the CCSD(T) curve, with very low RMSE and RMSPE values in the binding and tail regions.

The qualitative comparisons in Figures 3, 4, 5, 6 and the quantitative comparison on Table 5 indicate that the best four DFT or hybrid methods, for N_2_ on benzene, are *ω*B97X‐D, rev‐vdW‐DF2, B2PLYP‐D3, and B97D, in this order. The comparisons also indicate that all the hybrids, except MN15 and DSD‐BLYP, perform very well. The methods PBE‐D2, PBE‐XDM, vdW‐DF2, and RVV10 perform worse, but their RMSE and RMSPE values are reasonable.

### 3.3 Calculations of CO_2_ physisorbed on benzene: DFT‐based and hybrid methods versus CCSD(T) method

We have performed CCSD(T) calculations of CO_2_ on benzene, on the nine configurations depicted in Figure 1. According to CCSD(T) calculations, the most stable configuration of CO_2_ on benzene is the B_∥⊥_ one (see Table 6) with a binding energy of −0.0990 eV. This result compares favorably with those published in previous studies that provided CCSD(T)/CBS (Complete Basis Set) estimates of the binding energy of −0.1057^16^ and −0.1084 eV.^31^

**TABLE 6:** Equilibrium CO_2_‐benzene plane distances, *d*_*e*_, in Å, and binding energies, *E*_*b*_, in eV, for CO_2_ on different sites on benzene and with different orientations of the molecular axis, obtained with the CCSD(T) method, the counter poise method and the aug‐cc‐pVTZ basis set

Configuration	*d*_*e*_	*E*_*b*_
A_∥∥_	3.4	−0.0802
A_∥⊥_	3.3	−0.0889
A_⊥_	5.0	−0.0273
B_∥∥_	3.4	−0.0846
B_∥⊥_	3.3	−0.0990
B_⊥_	5.0	−0.0282
H_∥∥_	3.5	−0.0919
H_∥⊥_	3.5	−0.0919
H_⊥_	4.9	−0.0343

We ran calculations of CO_2_ on benzene, in the configuration B_∥⊥_, using different first principles methods, to obtain the interaction energy curve. The corresponding CO_2_‐benzene plane equilibrium distances and the binding energies are given in Table 7. The RMS and RMSP errors are also given in that table. The binding energy of CO_2_ on benzene is about −0.1 eV.

**TABLE 7:** Equilibrium CO_2_‐benzene plane distances, *d*_*e*_, in Å, and binding energies, *E*_*b*_, in eV, for CO_2_ on benzene and in the configuration B_∥⊥_, obtained with different methods. RMSE_*m*_ and RMSE_*t*_ are in eV, and RMSPE_*m*_ and RMSPE_*t*_ are in % (see text for their meaning)

Method	*d*_*e*_	*E*_*b*_	RMSE_*m*_	RMSPE_*m*_	RMSE_*t*_	RMSPE_*t*_
CCSD(T)	3.3	−0.0990	0.0000	0	0.0000	0
DFSAPT	3.3	−0.0936	0.0049	7	0.0011	6
VWN5	3.0	−0.1407	0.0343	58	0.0077	48
PW91	3.6	−0.0444	0.0584	84	0.0045	36
PBE	3.6	−0.0315	0.0708	102	0.0098	53
revPBE	4.3	−0.0123	0.1317	192	0.0121	52
B97D	3.3	−0.0827	0.0160	25	0.0029	17
B97‐D3	3.3	−0.1050	0.0095	15	0.0051	23
PBE‐D2	3.2	−0.1009	0.0046	8	0.0010	9
PBE‐D3	3.4	−0.1030	0.0089	14	0.0029	11
PBE‐TS	3.3	−0.1138	0.0111	15	0.0025	21
PBE‐XDM	3.4	−0.0969	0.0099	17	0.0030	29
MN15	3.2	−0.1120	0.0110	17	0.0080	50
*ω*B97xD	3.3	−0.0893	0.0119	20	0.0005	4
*ω*B97X‐D3BJ	3.3	−0.1113	0.0106	15	0.0004	3
*ω*B97X‐V	3.2	−0.1065	0.0058	7	0.0009	4
*ω*B97M‐D3BJ	3.2	−0.1059	0.0065	10	0.0005	5
*ω*B97M‐V	3.4	−0.1080	0.0083	13	0.0010	6
B2PLYP‐D3	3.3	−0.1012	0.0013	2	0.0006	3
DSD‐BLYP	3.3	−0.0819	0.0151	21	0.0044	21
DSD‐BLYP‐D3BJ	3.3	−0.1006	0.0026	4	0.0008	3
vdW‐DF	3.5	−0.1133	0.0270	45	0.0138	75
vdW‐DF‐C09	3.3	−0.1110	0.0159	25	0.0103	64
vdW‐DF‐cx	3.4	−0.1057	0.0178	29	0.0117	64
optB86b‐vdW	3.3	−0.1158	0.0185	27	0.0097	60
optB88‐vdW	3.3	−0.1147	0.0157	22	0.0073	52
vdW‐DF2	3.4	−0.1086	0.0120	19	0.0028	18
rev‐vdW‐DF2	3.3	−0.0920	0.0065	10	0.0007	8
vdW‐DF2‐C09	3.5	−0.0548	0.0401	56	0.0021	10
RVV10	3.3	−0.1056	0.0056	8	0.0021	29

**FIGURE 7:**
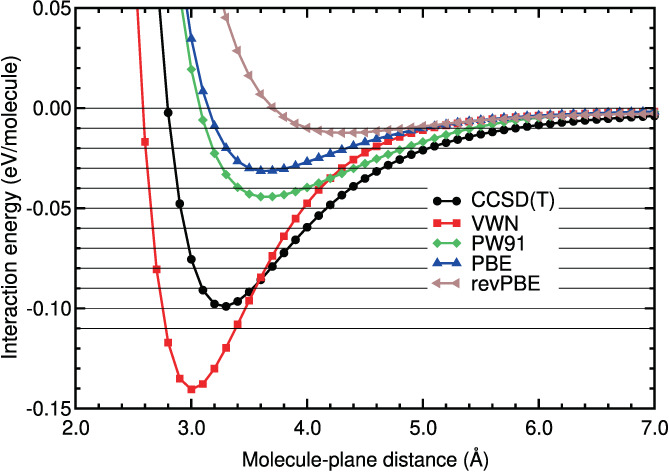
Interaction energy between CO_2_ and benzene as a function of the CO_2_‐benzene plane distance, obtained in CCSD(T), LDA, and GGA calculations

We discuss now the performance of the different DFAs assessed in this work for the CO_2_‐benzene system. The results are similar to those discussed previously for the N_2_‐benzene system. Thus, the energy curves of the LDA and GGA methods are very different from the CCSD(T) energy curve (see Figure 7 and Table 7). The RMSE and RMSPE values of the LDA and GGA curves are high or very high in both regions: The lowest RMSE and RMSPE in the binding region are 0.0343 eV and 58%, respectively, and the lowest RMSE and RMSPE in the tail region are 0.0045 eV and 36%, respectively.

**FIGURE 8:**
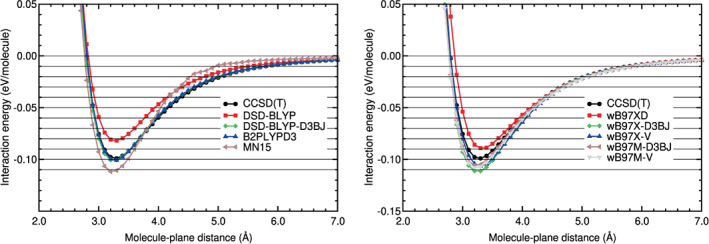
Interaction energy between CO_2_ and benzene as a function of the CO_2_‐benzene plane distance, obtained in CCSD(T) and hybrid calculations

Most of the hybrid method energy curves are much closer to the CCSD(T) energy curve (see Figure 8). As a matter of fact, the B2PLYPD3 and DSD‐BLYP‐D3BJ curves are almost indistinguishable from the CCSD(T) energy curve: Their RMSE and RMSPE values are very low in both regions. The relative errors, RMSPE, of these functionals are 2%–4% in the binding region and 3% in the tail region.

The *ω*B97XD and the other *ω*B97 energy curves are a bit different from the CCSD(T) curve in the binding region, but their RMSE and RMSPE values are reasonable. In the tail region the *ω*B97 energy curves are very similar to the CCSD(T) curve: Very low RMSE values, between 0.0004 and 0.0010 eV, and also very low RMSPE values, between 3% and 6% in the tail region. This shows that these DFAs describe correctly the long tail of the potential energy. The DSD‐BLYP curve has a regular performance (RMSPEs of 21% in both regions). The MN15 curve has a reasonable performance in the binding region (a RMSPE of 17%) and a poor performance in the tail region, with a high relative error: A RMSPE of 50%.

**FIGURE 9:**
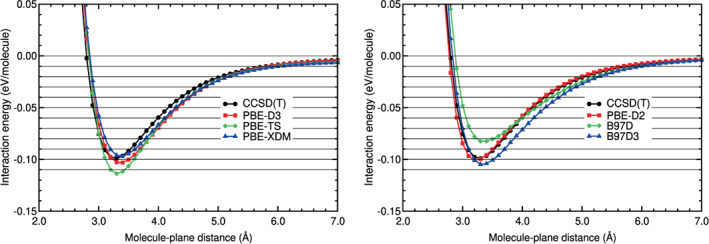
Interaction energy between CO_2_ and benzene as a function of the CO_2_‐benzene plane distance, obtained in CCSD(T) and DFT‐D calculations

The agreement of the DFT‐D energy curves with the CCSD(T) curve in the binding and tail regions is similar (see Figure 9 and Table 7). Their performance is, in general, reasonable in both regions: The RMSEs of this group of DFAs in the binding region are between 0.0046 and 0.0160 eV and the RMSPEs are between 8% and 25%; in the tail region the RMSEs are between 0.0010 and 0.0051 eV and the RMSPEs between 9% and 29%. The B97D seems to underestimate the binding region by some amount (RMSPE of 25%). The PBE‐XDM functional underestimate a little the binding region. The other semi‐empirical functionals overestimate a little the binding energy and region.

**FIGURE 10:**
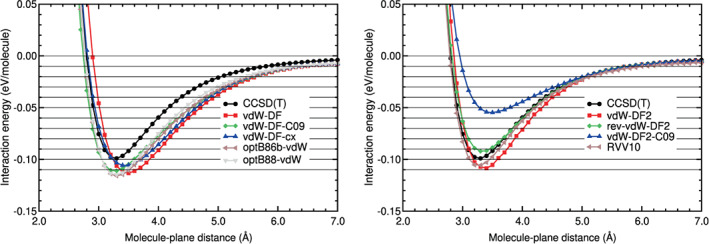
Interaction energy between CO_2_ and benzene as a function of the CO_2_‐benzene plane distance, obtained in CCSD(T), vdW‐DF, vdW‐DF2, and RVV10 calculations

As regards to the vdW‐DF methods (see Figure 10), they are far from the CCSD(T) curve, as in the N_2_‐benzene system. Their performance is worst than the performance of the DFT‐D methods. In the binding region the RMSE values are high, between 0.0157 and 0.0270 eV, and the RMSPEs are regular or high, between 22% and 45%. The RMSEs and RMSPEs are high in the tail region: Between 0.0773 and 0.0138 eV, and between 52% and 75%, respectively. It is interesting to note (see Table 7) that all the vdW‐DF functionals overestimate the binding energy and the binding region by a large or medium size fraction: Relative errors between 22% and 45% in that region.

The vdW‐DF2 methods perform well or very well in the tail region: The RMSEs are between 0.0007 and 0.0028 eV and RMSPEs are between 8% and 29%. There is not a general trend in the binding region. It can be noticed in Figure 10 that the vdW‐DF2‐C09 curve underestimates strongly the binding region energies. The extension of the underestimation is large, according to Table 7: RMSE and RMSPE of 0.0401 eV and 56%, respectively. The vdW‐DF2 curve has reasonable RMSE and RMSPE values in the binding regions. The RVV10 and rev‐vdW‐DF2 curves have low RMSE and RMSPE values in the binding regions.

The best methods for CO_2_ on benzene (they have the lowest values of RMSE and RMSPE in Table 7) are, in this order, B2PLYP‐D3, DSD‐BLYP‐D3BJ, *ω*B97x‐V, and PBE‐D2. The rest of the *ω*B97 functionals along with rev‐vdW‐DF2 and PBE‐D3 perform also reasonable well. Interestingly, RVV10 performs very well in the binding region, but its performance is mediocre in the long tail region.

Witte and co‐workers^16^ also found that PBE‐D, *ω*B97X, and vdW‐DF2 functionals perform well on benzene‐CO_2_. In particular, we point out that the *ω*B97X functionals perform almost as well as the double‐hybrids tested in this study, which is an interesting conclusion since the later involved a much larger computational cost. For this particular system, PBE‐D2 also provides a similar accuracy to the *ω*B97X functionals. In any case, the main conclusion at this point is that the double‐hybrids are the best performers among the tested functionals for benzene‐CO_2_.

### 3.4 Symmetry adapted perturbation theory, DFSAPT

In order to shed some light on the nature of the bonding between benzene and both N_2_ and CO_2_, we have carried out a Density Functional–Symmetry Adapted Perturbation Theory (DFSAPT) analysis. Table 8 collects the individual contributions and the total interaction energies evaluated at the minimum of the CCSD(T) potential energy curves.

**TABLE 8:** DFSAPT components in eV of the interaction energies, Δ*E*_*int*_, for benzene‐N_2_ and benzene‐CO_2_ complexes

Component	Benzene‐N_2_	Benzene‐CO_2_
$E_{\text{elst}}^{\left(1\right)}$	−0.0281	−0.0640
$E_{\text{exch}}^{\left(1\right)}$	+0.0631	+0.1187
*E* ^(1)^	+0.0350	+0.0548
$E_{\mathrm{ind}}^{\left(2\right)}$	−0.0180	−0.0587
$E_{\text{exch}-\mathrm{ind}}^{\left(2\right)}$	+0.0160	+0.0498
$E_{\text{disp}}^{\left(2\right)}$	−0.1001	−0.1553
$E_{\text{exch}-\text{disp}}^{\left(2\right)}$	+0.0084	+0.0158
*E* ^(2)^	−0.0937	−0.1484
Δ*E*_*int*_ = *E* ^(1)^ + *E* ^(2)^	−0.0587	−0.0936
*δ*(*HF*)	−0.0080	−0.0063
$\Delta E_{\mathrm{int}}^{\text{corr}}=\Delta E_{\mathrm{int}}+\delta \left(\mathrm{HF}\right)$	−0.0667	−0.0999
*E*_*b*_ CCSD(T)	−0.0608	−0.0990

Table 8 shows that the largest contributor to the stabilization of these complexes is the dispersion energy, which represents 68% and 56% of the total attractive contributions ($E_{\text{elst}}^{\left(1\right)}+E_{\mathrm{ind}}^{\left(2\right)}+E_{\text{disp}}^{\left(2\right)}$) for the N_2_ and CO_2_ complexes, respectively. Indeed, first‐order interactions ($E^{\left(1\right)}=E_{\text{elst}}^{\left(1\right)}+E_{\text{exch}}^{\left(1\right)}$) are positive, meaning that electrostatic forces alone cannot hold these systems bound. Induction contributions ($E_{\mathrm{ind}}^{\left(2\right)}+E_{\text{exch}-\mathrm{ind}}^{\left(2\right)}$) are small and also positive for both complexes. Thus, DFSAPT results show that, as expected, these systems are mainly bound by dispersion forces.

The total first‐ and second‐order components *E* ^(1)^ + *E* ^(2)^ yield interaction energies of −0.0587 and −0.0936 eV for N_2_ and CO_2_, respectively, a little smaller than the reference CCSD(T) values, −0.0608 and −0.0990 eV, respectively. As pointed out in the methods sections, higher that second‐order contributions can be estimated by means of the *δ*(*HF*) term. In these systems, *δ*(*HF*) represents a small percentage of the total interaction energy (12% and 7% for N_2_ and CO_2_, respectively) suggesting that first‐ and second‐order terms are clearly the most important contributors. Including higher‐order terms, the DFSAPT interaction energy is −0.0667 and −0.0999 eV, for N_2_ and CO_2_, respectively, slightly larger than the reference values. For N_2_ the uncorrected energy is closer to the reference; in the case of CO_2_, the corrected one is better. In any case, both of them are within 0.007 eV of the CCSD(T) value, showing that DFSAPT provides very accurate interaction energies for these systems.

### 3.5 Calculations of N_2_ physisorbed on graphene: DFT‐based methods versus experimental data

Calculations of the nine configurations of N_2_ on graphene, plotted in Figure 2, have been carried out using the rev‐vdW‐DF2 functional, to find out the most stable configuration. The results are in Table 9. The most stable configuration is the configuration H_∥⊥_. The interaction energy and the binding energy are defined in Equation (1), with molecule = N_2_ and *A* = graphene.

**TABLE 9:** Equilibrium N_2_‐graphene plane distances, *d*_*e*_, in Å, and binding energies, *E*_*b*_, in eV, for N_2_ on different sites on graphene and with different orientations of the molecular axis, obtained with the rev‐vdW‐DF2 method

Configuration	*d*_*e*_	*E*_*b*_
A_∥∥_	3.3	−0.1084
A_∥⊥_	3.3	−0.1084
A_⊥_	3.8	−0.0780
B_∥∥_	3.3	−0.1114
B_∥⊥_	3.3	−0.1091
B_⊥_	3.8	−0.0793
H_∥∥_	3.3	−0.1173
H_∥⊥_	3.3	−0.1175
H_⊥_	3.7	−0.0915

**FIGURE 11:**
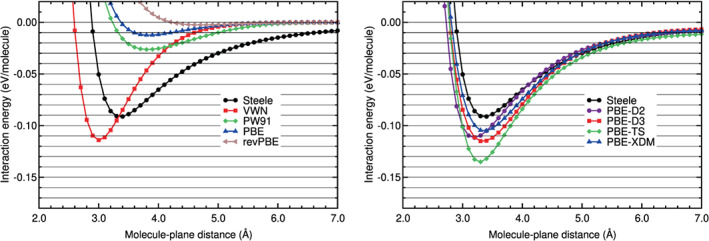
Interaction energy between N_2_ and graphene as a function of the N_2_‐graphene plane distance, obtained with LDA, GGA, and DFT‐D methods. N_2_ is in the configuration H_∥⊥_

**FIGURE 12:**
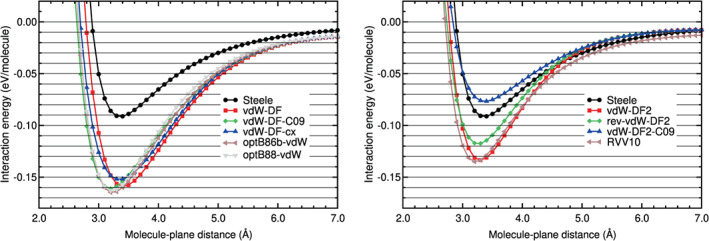
Interaction energy between N_2_ and graphene as a function of the N_2_‐graphene plane distance, obtained with vdW‐DF and vdW‐DF2 methods and the RVV10 method. N_2_ is in the configuration H_∥⊥_

Calculations of the interaction energy of N_2_ physisorbed on graphene, on the configuration H_∥⊥_, have been carried out with different DFT‐based methods. The corresponding interaction energy curves are plotted in Figures 11 and 12. The black horizontal lines in Figures 11 and 12 have the purpose to guide the eye in the comparison of the different curves. The Steele potential energy curve of N_2_ on graphene is also plotted on those figures (see the black solid line and the black circles on the mentioned figures). That potential is based on experimental data, as it was explained in a former section. The Steele potential energy curve is the reference curve for N_2_ on graphene and all the DFT energy curves will be compared with that.

The plot of the interaction energy curves serves as a qualitative comparison. A quantitative comparison with the Steele potential energy has been also made, by means of the calculation of the RMS and RMSP errors, using as pivot or reference the Steele potential energy curve of N_2_ on graphene. The errors, equilibrium distances and binding energies obtained with several DFAs are in Table 10. The intervals of molecule‐plane distances of the minimum and tail region of N_2_ on graphene, used to calculate the errors, are in Table 2.

**TABLE 10:** Equilibrium N_2_‐graphene plane distances, *d*_*e*_, in Å, and binding energies, *E*_*b*_, in eV, for N_2_ on the configuration H_∥⊥_, obtained with different methods. RMSE_*m*_ and RMSE_*t*_ are in eV, and RMSPE_*m*_ and RMSPE_*t*_ are in % (see text for their meaning)

Method	*d*_*e*_	*E*_*b*_	RMSE_*m*_	RMSPE_*m*_	RMSE_*t*_	RMSPE_*t*_
Steele	3.4	−0.0912	0.0000	0	0.0000	0
VWN‐RPA	3.0	−0.1140	0.0308	52	0.0201	91
PW91	3.8	−0.0263	0.0653	92	0.0168	82
PBE	3.8	−0.0125	0.0786	111	0.0215	93
revPBE	4.7	−0.0025	0.1286	184	0.0230	96
PBE‐D2	3.2	−0.1117	0.0194	32	0.0022	10
PBE‐D3	3.3	−0.1150	0.0211	30	0.0026	16
PBE‐TS	3.3	−0.1352	0.0339	47	0.0042	21
PBE‐XDM	3.4	−0.1050	0.0125	18	0.0020	11
vdW‐DF	3.4	−0.1590	0.0614	86	0.0196	71
vdW‐DF‐C09	3.2	−0.1611	0.0623	90	0.0163	63
vdW‐DF‐cx	3.3	−0.1519	0.0586	84	0.0175	64
optB86b‐vdW	3.2	−0.1646	0.0639	92	0.0156	60
optB88‐vdW	3.2	−0.1631	0.0605	87	0.0129	53
vdW‐DF2	3.3	−0.1335	0.0335	47	0.0030	15
rev‐vdW‐DF2	3.3	−0.1175	0.0229	35	0.0033	18
vdW‐DF2‐C09	3.4	−0.0767	0.0106	14	0.0038	18
RVV10	3.2	−0.1351	0.0358	53	0.0049	29

The comparison of the DFT interaction energy curves with the Steele interaction energy curve (Figures 11 and 12 and Table 10) reveals several findings. Similar to the benzene case, the LDA and GGA functionals perform very badly. VWN overestimates the binding energy and the GGA functionals underestimate that energy. The RMSE and RMSPE values in the binding and tail regions are very high. The smallest RMSE and RMSPE are 0.0308 eV and 52% and, they correspond to the VWN functional and to the binding region.

The semiempirical methods perform very well in the tail region: Their RMSE and RMSPE values are low, between 0.0020 and 0.0042 eV and between 10% and 22%, respectively (see Table 10). In the binding region, the PBE‐XDM functional performs reasonably well, the PBE‐D2 and PBE‐D3 functionals perform regularly and the performance of the PBE‐TS functional is poor. The vdW‐DF group of functionals has a very poor performance in the binding and tail regions: The RMSE and RMSPE values are high; the lowest RMSE and RMSPE are 0.0129 eV and 53%, respectively.

As regards to the vdW‐DF2 group of functionals, they agree well with the Steele energy curve in the tail region: The RMSE is in the range 0.0030–0.0049 eV and the RMSPE in the range 15%–29%. However, in the binding region, only the vdW‐DF2‐CO9 functional performs reasonably well (RMSE = 0.0106 eV and RMSPE = 14%) and the other functionals have a regular or poor performance (RMSEs in the interval 0.0229–0.0358 eV and RMSPEs in the interval 35%–53%). The vdW‐DF2‐C09 underestimates a little the binding region and the other vdW‐DF2 functionals overestimates by a large factor the binding region, as can be noticed in Figure 12 and in Table 10.

Among the DFAs studied for N_2_ on graphene, the PBE‐XDM and the vdW‐DF2‐C09 functionals have a good or reasonable agreement with the Steele interaction energy curve in both regions and the PBE‐D2, PBE‐D3 and rev‐vdW‐DF2 have a regular agreement, according to the results plotted in Figures 11 and 12 and gathered in Table 10).

### 3.6 Calculations of CO_2_ physisorbed on graphene: DFT‐based methods versus experimental data

Calculations of the nine configurations of CO_2_ on graphene, plotted in Figure 2, have been carried out using the rev‐vdW‐DF2 functional, to find out the most stable configuration. The results are in Table 11. The most stable configuration for CO_2_ on graphene is the configuration B_∥⊥_. The interaction energy and the binding energy are defined in Equation (1), with molecule = CO_2_ and *A* = graphene.

**TABLE 11:** Equilibrium CO_2_‐graphene plane distances, *d*_*e*_, in Å, and binding energies, *E*_*b*_, in eV, for CO_2_ on different sites on graphene and with different orientations of the molecular axis, obtained with the rev‐vdW‐DF2 method

Configuration	*d*_*e*_	*E*_*b*_
A_∥∥_	3.2	−0.1603
A_∥⊥_	3.3	−0.1596
A_⊥_	4.3	−0.0854
B_∥∥_	3.3	−0.1527
B_∥⊥_	3.2	−0.1689
B_⊥_	4.3	−0.0863
H_∥∥_	3.3	−0.1508
H_∥⊥_	3.3	−0.1509
H_⊥_	4.2	−0.0925

**FIGURE 13:**
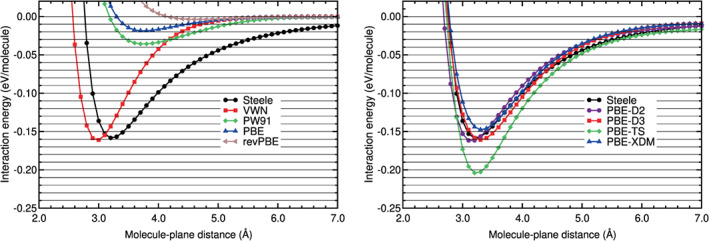
Interaction energy between CO_2_ and graphene as a function of the CO_2_‐graphene plane distance, obtained with LDA, GGA, and DFT‐D methods. CO_2_ is in the configuration B_∥⊥_

**FIGURE 14:**
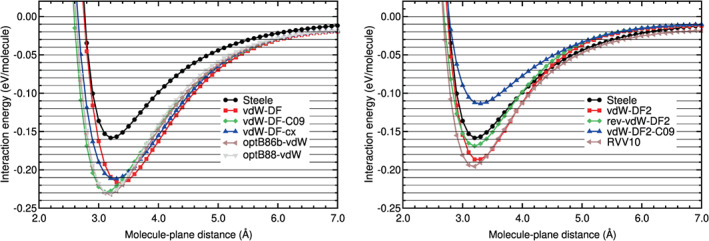
Interaction energy between CO_2_ and graphene as a function of the CO_2_‐graphene plane distance, obtained with vdW‐DF and vdW‐DF2 methods and the RVV10 method. CO_2_ is in the configuration B_∥⊥_

The interaction energy curve of CO_2_ physisorbed on graphene, on the configuration B_∥⊥_, has been calculated using different DFT‐based methods. These energy curves and the Steele potential energy curve of CO_2_ on graphene are plotted in Figures 13 and 14. The Steele potential energy curve was obtained from experimental data, as it was explained in a former section. The RMS and RMSP errors, and the equilibrium distances and binding energies are collected in Table 12. The errors are defined in Equations (2) and (3), with molecule = CO_2_ and *A* = graphene and using as reference the Steele potential energy curve. The intervals of molecule‐plane distances of the minimum and tail region of CO_2_ on graphene, used to calculate the errors, are described in Table 2.

**TABLE 12:** Equilibrium CO_2_‐graphene plane distances, *d*_*e*_, in Å, and binding energies, *E*_*b*_, in eV, for CO_2_ on the configuration B_∥⊥_, obtained with different methods. RMSE_*m*_ and RMSE_*t*_ are in eV, and RMSPE_*m*_ and RMSPE_*t*_ are in % (see text for their meaning)

Method	*d*_*e*_	*E*_*b*_	RMSE_*m*_	RMSPE_*m*_	RMSE_*t*_	RMSPE_*t*_
Steele	3.2	−0.1584	0.0000	0	0.0000	0
VWN‐RPA	3.0	−0.1611	0.0437	38	0.0337	90
PW91	3.8	−0.0357	0.1305	101	0.0300	81
PBE	3.8	−0.0185	0.1476	114	0.0378	93
revPBE	4.6	−0.0037	0.2294	178	0.0417	96
PBE‐D2	3.1	−0.1616	0.0115	10	0.0062	15
PBE‐D3	3.3	−0.1609	0.0075	6	0.0052	22
PBE‐TS	3.2	−0.2040	0.0368	27	0.0066	19
PBE‐XDM	3.3	−0.1476	0.0128	10	0.0066	24
vdW‐DF	3.4	−0.2173	0.0588	46	0.0267	54
vdW‐DF‐C09	3.1	−0.2287	0.0661	53	0.0201	44
vdW‐DF‐cx	3.3	−0.2117	0.0576	46	0.0232	46
optB86b‐vdW	3.2	−0.2320	0.0689	54	0.0200	43
optB88‐vdW	3.2	−0.2308	0.0658	51	0.0165	38
vdW‐DF2	3.2	−0.1865	0.0237	17	0.0054	17
rev‐vdW‐DF2	3.2	−0.1689	0.0112	9	0.0070	22
vdW‐DF2‐C09	3.3	−0.1137	0.0369	28	0.0099	24
RVV10	3.2	−0.1955	0.0327	26	0.0054	23

An analysis of the results (Figures 13 and 14 and Table 12) indicates some similarities with the results of N_2_ on graphene and also some differences. The LDA and GGA interaction energy curves are very different from the Steele energy curve in all the regions of the curve, as in the case of N_2_ on graphene: The RMSE and RMSPE are very high; the lowest RMSE is 0.0300 eV and the lowest RMSPE is 38%. GGA functionals underestimate strongly the binding energy. The VWN functional, however, yields a binding energy similar to that of the Steele energy curve. The VWN energy curve is still very different from the Steele potential energy curve: Its RMSPE is 38% and 90% on the binding and tail regions, respectively.

Most of semiempirical methods, except PBE‐TS, perform well or reasonably well in both regions of the curve. Their RMSEs are low in the binding region, between 0.0075 and 0.0128 eV, and 0.0052 and 0.0066 eV in the tail region (see Table 12). Their relative errors, RMSPEs, are also low in the binding region, between 6% and 10%, and a little higher in the tail region, between 15% and 24%. The PBE‐TS overestimates the binding energy by a large factor. The other functionals yield binding energies close to the binding energy of the Steele potential.

The performance of the vdW‐DF functionals is very poor, as in the case of N_2_ on graphene. They overestimate the interaction energies in all the points of the interaction energy curve. The relative errors of this group of functionals is about 50% in both regions of the energy curve.

The vdW‐DF2 functionals agree reasonably well with the Steele energy curve in the tail region. As regards to the binding region, the rev‐vdW‐DF2 functional performs well (RMSE 0.0112 and RMSPE 9%) and the performance of the other functionals is regular or very poor in the binding region (RMSEs in the range 0.0237–0.0369 eV and RMSPEs in the range 17%–28%). The vdW‐DF2‐C09 functional underestimates the binding energy and the binding region by a large factor, the RVV10 functional overestimates the binding region also by a large factor (RMSPE of 26%) and the vdW‐DF2 overestimates the binding region by a moderate factor (RMSPE of 17%).

Taking into account the results in both regions of the energy curve of CO_2_ on graphene, the functionals with a good or reasonable performance (RMSPE ≤ 10% and ≤24% in the binding and tail region, respectively) for CO_2_ on graphene are: PBE‐D2, PBE‐D3, PBE‐XDM, vdW‐DF2 and rev‐vdW‐DF2.

## 4 SUMMARY AND CONCLUSIONS

The interaction energy curves of N_2_ and CO_2_ on benzene and graphene have been calculated by means of different methods: CCSD(T), double hybrid, hybrid and DFAs. The energy curves on benzene have been compared with the CCSD(T) energy curve, and the energy curves on graphene with the Steele potential energy curve obtained from experimental data.

Some general trends of the DFAs used for the four systems (N_2_‐benzene, CO_2_‐benzene, N_2_‐graphene, and CO_2_‐graphene) can be noticed. The overestimation of the binding energy and region by the LDA functionals and the underestimation by the GGA functionals observed in the present calculations of the four systems, have been also observed in many physisorbed systems. The group of vdW‐DF functionals underestimates the binding energy and the energies of the binding and tail region of the interaction energy curves of the four systems studied in this work. A similar underestimation of the binding and/or the interaction energies, but not identical, was also reported for another systems. Kocman et al.^32^ calculated and studied the energy curves of H_2_ on coronene and coronene modified with boron and lithium using different DFAs and the CCSD(T) method. They also obtained that vdW‐DF functionals overestimate the binding energy and the energies in the binding and tail regions of the interaction energy curve. The vdW‐DF functionals also underestimate part of the binding region and all the tail region of the interaction energy curve of H_2_ on benzene, when compared with the CCSD(T) interaction energy curve.^15^ However, the vdW‐DF functionals yield binding energies close to the CCSD(T) binding energy of H_2_ on benzene. These functionals underestimate by a large factor the adsorption energies of the lowest level of H_2_ on graphene, compared to the experimental value of the adsorption energy.^15^

The vdW‐DF2‐C09 functional underestimates the binding energy and the energies around the binding region in the four systems studied. In the case of N_2_ on graphene, the binding energy and region are underestimated, but only by a small factor, and there is a good agreement between the vdW‐DF2‐C09 energy curve and the experimental Steele potential energy curve in the binding and tail region. In the other three systems studied, the underestimation of the energies is large in the binding region. The vdW‐DF2‐C09 also underestimates the binding energy and region of H_2_ on benzene by a large factor.^15^ This functional underestimates also by a large factor the adsorption energy of the lowest level of H_2_ on graphene, compared to the experimental value of the adsorption energy.^15^ Finally, the vdW‐DF2 and RVV10 functionals overestimate the binding energy and the binding energy region in the four systems studied.

When comparing atom centered basis set results for both systems, benzene‐N_2_ and benzene‐CO_2_, with the reference CCSD(T) calculations, we find that the best agreement is found among double hybrids with dispersion corrections and *ω*B97 based DFAs. In the short or binding region of the interaction energy curve of these molecules on benzene, double hybrids with dispersion corrections slightly outperform *ω*B97 functionals for benzene‐CO_2_, whereas for benzene‐N_2_ both set of functionals perform equally well. On the other hand, in the long or tail region of the interaction energy curve, both double hybrids with dispersion corrections and *ω*B97 functionals provide similar accuracy. It is worth to point out that, according to the present results of N_2_ and CO_2_ on benzene, the dispersion corrections must be added to the double hybrids, in order to reproduce the CCSD(T) curves accurately. More specifically, DSD‐BLYP (without dispersion corrections) performs poorly in the long range region for both systems and must be used with caution.

Regarding the rest of tested DFAs, none of them achieve the same consistent accuracy for both systems (N_2_‐benzene and CO_2_‐benzene). Nevertheless, we would like to highlight the PBE‐D2 and rev‐vdW‐DF2 functionals, which efficiently reproduce long range curves and can be recommended for studies focused on this region, like physisorption processes. DFSAPT, on the other hand, provides very accurate results compared to CCSD(T) and can be recommended both in the binding and long range regions. It must be pointed out that DFSAPT has a similar computational cost as a GGA functional.

The comparison of the interaction energy curves of N_2_ and CO_2_ on graphene obtained using several DFAs, with the Steele interaction energy curves, based on experimental data, indicate that only the PBE‐XDM functional has a good or reasonable agreement for both systems in the binding and tail regions. The PBE‐D2, PBE‐D3, and rev‐vdW‐DF2 functionals have a mediocre performance for N_2_ on graphene, but perform reasonably well for CO_2_ on graphene.

## Supporting information


**Appendix S1** Supporting Information.

## Data Availability

The data that support the findings of this study are available from the corresponding author upon reasonable request.
