# Supplementary material for: Assessment of density functional approximations for N2 and CO2 physisorption on benzene and graphene
Source: J Comput Chem. 2022 Jun 6;43(21):1403–19. doi: 10.1002/jcc.26945 (PMC9328377; doi:10.1002/jcc.26945)
Supplement: Supplementary file 1 — Appendix S1 Supporting Information. [file JCC-43-1403-s001.pdf]

Supporting Information

Assessment of Density Functional Approximations for N_2 and CO_2 Physisorption on Benzene and Graphene

V. M. Rayón[1,*] and I. Cabria[2,†]

[1]Departamento de Química Física y Química Inorgánica,

Facultad de Ciencias, Universidad de Valladolid, Valladolid

[2]Departamento de Física Teórica, Atómica y Óptica,

Facultad de Ciencias, Universidad de Valladolid, Valladolid

PACS numbers: 71.15.Mb,68.43.-h,88.30.R-

Keywords: Physisorption, Gas Storage, Dispersion Interactions, Graphene, DFT, Pore Size Distribution

I. GAUSSIAN 16

The Gaussian 16 (G16RevA.03) code[1] was used to make calculations of N_2 and CO_2 on benzene, but not on graphene. This code has several methods implemented and used in the present work: CCSD(T)[2], PBE[3,4], VWN5[5], PW91[6], B97D[7,8], PBE-D2[7] B97D3[7], B2PLYP-D3[9] and MN15[10,11] and ωB97-XD[12]. The VWN5 method uses the LDA for exchange and correlation effects with the Vosko-Wilk-Nusair (VWN5) parameterization[5]. PBE and PW91 use GGA functionals due to Perdew, Burke and Ernzerhof,[3,4] and to Perdew and Wang,[6] respectively. The PBE-D2 and B97D methods include the dispersion corrections according to the DFT-D scheme. PBE-D2 stands for the PBE functional plus the Grimme's D2 dispersion corrections[7]. B97D is a functional proposed by Grimme[7], based on the B97 functional of Becke[8] and the Grimme's D2 dispersion corrections. It is a reparameterization of the original B97 functional.

The G16 code performs all-electron calculations using gaussian basis sets to expand the wavefunctions. The basis set used for all the methods is the augmented correlation-consistent basis set, aug-cc-pVTZ, of Dunning *et al.*[13–17]. Basis sets of the aug-cc-pVXZ family contain diffuse functions which are necessary to account for dispersion interactions. The spin restricted calculations (because N_2, CO_2 and benzene are closed-shell systems) were done self-consistently with a total energy convergence tolerance of 2.72×10^{-5} eV. The optimizations of the geometries were run until the forces on the atoms were lower than 4.63×10^{-3} eV/Å and the displacements of atoms were lower than 1.9×10^{-4} Å.

A test of the basis set is provided in Figure 1, where a comparison of CCSD(T) calculations of N_2 interacting with benzene with the para-para orientation on top of the center of the benzene hexagon, done with the aug-cc-pVTZ and aug-cc-pVQZ basis sets is presented. The counterpoise method was used to calculate the interaction energies in Figure 1. The aug-cc-pVQZ basis set is the largest one. The difference between the aug-cc-pVTZ and aug-cc-pVQZ interaction energies is smaller than 10^{-3} eV/molecule in the region near the minimum, and it becomes negligible at larger N_2-benzene distances. The CCSD(T) interaction energies obtained with the aug-cc-pVQZ basis set are very similar to those obtained using the aug-cc-pVTZ basis set (See Fig. 1), but the computational cost was much higher.

Therefore, the aug-cc-pVTZ basis set and the counterpoise method have been selected to make the G16 calculations with all the methods.

The use of aug-cc-pVTZ basis sets, the correction of the BSSE and the value of the self-consistent threshold used mean that the present G16 calculations have a high degree of precision.

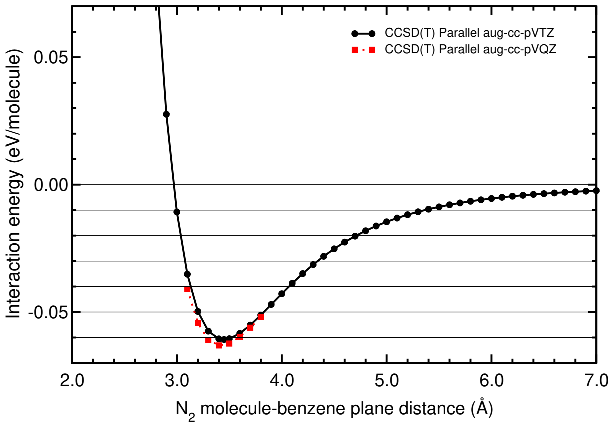

FIG. 1: (Color online) Interaction energy between N_2 and benzene as a function of the N_2-benzene distance, obtained by the CCSD(T) method and the counterpoise method, as implemented in the Gaussian 16 code. Calculations performed with the aug-cc-pVTZ and aug-cc-pVQZ basis sets are compared. The molecule is on top of the center of the benzene molecule and with the orientation $\|\|$.

A comparison of the interaction energy curve obtained with the CCSD(T) method, the counterpoise method and the aug-cc-pVTZ basis, was carried out for the N_2 molecule on the center of the hexagon and with the three main orientations of the N_2 axis. The results are plotted in Fig. 2. As can be noticed, the difference between the orientations $\|\|$ and $\|\perp$ are very small, and close to the convergence threshold, 0.00001 eV.

II. ORCA

Orca 4.2.1[18,19] was used to perform the ωB97X-D3BJ[20], ωB97X-V[21], ωB97M-D3BJ[20] and ωB97M-V[22] calculations. The first two DFAs are reparametrizations of the ωB97X-D[12] functional with the DFT-D3 correction[23] with zero-damping (ωB97X-D3BJ) and with the non-

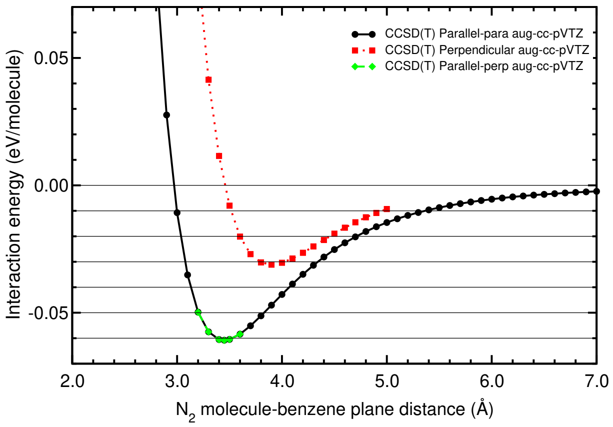

FIG. 2: (Color online) Interaction energy between N_2 and benzene as a function of the N_2-benzene distance, obtained by the CCSD(T) method and the counterpoise method, as implemented in the Gaussian 16 code, with the aug-cc-pVTZ basis set. The molecule is on top of the center of the benzene molecule and on the three main orientations.

local van der Waals VV10 kernel (ωB97X-V), respectively. All functionals were treated with the chain-of-sphere approximation for the evaluation of the exchange integrals (RIJCOSX)[24]. We have checked, for the particular case of N_2–benzene with the ωB97X-D3BJ functional, that the RIJCOSX treatment introduces negligible differences in geometries and interaction energies when compared to the exact calculation of exchange integrals. Besides, standard defaults in Orca were used, except: (1) SCF convergence criterion was set to 'tightscf", (2) quadrature grid used was '4" and the final grid was turned off, 'nofinalgrid". The aug-cc-pVTZ basis set was used with an automatically constructed auxiliary basis set for the simultaneous fitting of Coulomb and exchange calculations, 'autoaux".

III. MOLPRO

DFSAPT[25,26] calculations were carried out with the Molpro 2018.2 code[27–29]. We have employed a dimer centered aug-cc-pVTZ basis set (DBCS) (that is, monomers properties are calculated using the whole basis set of the dimer). The PBE0AC(BRJ)[30] potential with automatic asymptotic corrections was used. The following global thresholds applied to the DFSAPT calculations (atomic units): energy (1×10^{-8}), orbitals (1×10^{-5}), grid (1×10^{-8}). Finally, density fitting was employed to approximate the integrals in DFSAPT. The default

aug-cc-pVTZ/JKFIT and aug-cc-pVTZ/MP2FIT fitting basis sets were used.

IV. QUANTUM ESPRESSO

The Quantum Espresso, QE, code, version 6.3, was used to make calculations of N_2 and CO_2 physisorbed on benzene with the functionals PBE-TS[31], PBE-XDM[32,33], vdW-DF[34], optB88-vdW[35], optB86b-vdW[36], vdW-DF-cx[37], vdW-DF-C09[38], vdW-DF2[39], vdW-DF2-C09[40], rev-vdW-DF2[41] and RVV10[42]. The QE code was also used to make calculations of N_2 and CO_2 physisorbed on graphene with the functionals VWN[5] (the VWN-RPA), PBE[3,4], PBE-D2[7], PBE-D3[43], PBE-TS, PBE-XDM, vdW-DF, optB88-vdW, optB86b-vdW, vdW-DF-cx, vdW-DF-C09, vdW-DF2, vdW-DF2-C09, rev-vdW-DF2 and RVV10. The works of Thonhauser *et al.* and Román-Pérez and Soler are relevant in the practical implementation of the vdW-DF functionals[44,45]. This code uses planewaves basis sets. The kinetic energy cutoff for the wavefunctions was set at 120 Ry and the kinetic energy cutoff for charge density and potential was set at 480 Ry, after carrying out tests of the convergence of the interaction energy with respect to the cutoffs. With these values of the cutoffs, the precision of the interaction energies $E(d)$ is 10^{-4} eV. All the calculations were performed using the Martins-Troullier norm-conserving pseudopotentials[46], except the PBE-XDM calculations, which were performed using the Kresse-Joubert Projector Augmented-Wave, PAW, pseudopotentials[47]. Only the Γ point was used in the calculations of both, benzene and graphene.

The unit cell for the benzene calculations, using the QE code, was a cubic supercell with a lattice parameter of 15 Å. The unit cell for the graphene calculations, using the QE code, was a hexagonal planar layer of 72 carbon atoms, with a lattice parameter of 15 Å in the direction perpendicular to the graphene plane. The lattice parameter of 15 Å for benzene and graphene was chosen after carrying tests of the convergence of the interaction energy with respect to the lattice parameter. The SCF procedure was considered converged if the absolute total energy change was below 1.36×10^{-6} eV for benzene calculations and 1.36×10^{-7} eV for graphene calculations. The geometries were considered converged when the forces on the atoms were less than 2.57×10^{-4} eV/Å. The calculations of the free molecules

were performed in the same supercell and conditions.

V. OPTIMIZATION OF THE GEOMETRIES OF N_2, CO_2, BENZENE AND GRAPHENE

The results of the optimization of the geometries of N_2, CO_2, benzene and graphene carried out with the CCSD(T) method and with different functionals are reported in Table I. The theoretical or experimental references for the geometries are at the bottom of that table. The experimental equilibrium N-N distance in the N_2 molecule and the experimental equilibrium C-O distance in the CO_2 molecule are 1.0975 and 1.1600 Å, respectively[48,49]. Experimental values of the geometry of benzene, d_{C-C}=1.3902 ± 0.0002 Å and d_{C-H}=1.0862 ± 0.0015 Å, were obtained from Plíva et al.[50]. The experimental carbon-carbon distance in graphite is 1.42 Å[51].

All the tested functionals describe correctly the equilibrium geometries of the monomers. As a matter of fact, most of them are within 0.0060 Å from the experimental values. The mean unsigned averaged errors (MUAE) are 0.0040, 0.0054, 0.0030, and 0.0036 Å for N–N, C–O, C–C, and C–H, respectively. All the DFAs families perform well in this regard. Indeed, there is no family which performs better than the others for the four bonds: for example, hybrids and double hybrids yield the lowest MUAE for C–C (0.0016 Å) and C–O (0.0025 Å), but the largest MUAE for N–N (0.0053 Å) and C–H (0.0054 Å), while local and GGA DFAs are the best performers for N–N and C–H. All the DFAs used for graphene perform extremely well with MUAE 0.0016 Å and a maximum error of just 0.0045 Å (PBE-XDM). The optimized geometries of each corresponding method were kept frozen and used later to calculate the interaction of N_2 and CO_2 on benzene and graphene.

Acknowledgments: This work was supported under MICINN research projects from Spain (Grants PGC2018-093745-B-I00 and PID2020-117742GB-I00), *Junta de Castilla y León* (Grant VA124G18) and the University of Valladolid, Spain. The facilities provided by *Centro de Proceso de Datos - Parque Científico* of the University of Valladolid are also

TABLE I: Equilibrium N–N distance in N_2, C–O distance in CO_2, C–C and C–H distances in benzene and C–C distance in graphene, in Å, obtained with different theoretical methods

Method	d_{N-N}	d_{C-O}	d_{C-C} benzene	d_{C-H} benzene	d_{C-C} graphene
CCSD(T)	1.1040	1.1670	1.3980	1.0839	–
VWN	1.0958	1.1633	1.3858	1.0937	1.4169
PW91	1.1015	1.1706	1.3959	1.0890	1.4188
PBE	1.1031	1.1720	1.3974	1.0909	1.4196
revPBE	1.0958	1.1651	1.3926	1.0842	1.4204
B97D	1.0992	1.1686	1.3988	1.0873	–
B97-D3	1.0992	1.1683	1.3962	1.0863	–
PBE-D2	1.1031	1.1721	1.3982	1.0910	1.4195
PBE-TS	1.0958	1.1650	1.3917	1.0852	1.4180
PBE-XDM	1.1063	1.1710	1.3963	1.0905	1.4245
PBE-D3	1.0958	1.1650	1.3919	1.0853	1.4194
MN15	1.0927	1.1590	1.3893	1.0816	–
ωB97x-D	1.0882	1.1565	1.3872	1.0816	–
ωB97X-D3BJ	1.0913	1.1602	1.3904	1.0839	–
ωB97M-D3BJ	1.0900	1.1589	1.3871	1.0798	–
B2PLYP-D3	1.0995	1.1647	1.3919	1.0807	–
DSD-BLYP	1.1010	1.1631	1.3904	1.0785	–
DSD-BLYP-D3BJ	1.1011	1.1642	1.3921	1.0798	–
vdW-DF	1.0917	1.1629	1.3909	1.0797	1.4197
vdW-DF-C09	1.0957	1.1650	1.3906	1.0884	1.4180
vdW-DF-cx	1.0957	1.1651	1.3908	1.0879	1.4181
optB86b-vdW	1.0947	1.1645	1.3906	1.0863	1.4183
optB88-vdW	1.0923	1.1629	1.3895	1.0825	1.4178
vdW-DF2	1.0909	1.1632	1.3926	1.0772	1.4224
rev-vdW-DF2	1.0947	1.1647	1.3910	1.0864	1.4186
vdW-DF2-C09	1.0957	1.1653	1.3912	1.0882	1.4185
RVV10	1.0951	1.1651	1.3935	1.0825	1.4218
Reference	1.0975[48,49]	1.1600[48,49]	1.3902[50]	1.0862[50]	1.42[51]

acknowledged.

---

* Electronic address: victormanuel.rayon@uva.es

† Electronic address: ivan.cabria@uva.es

[1] M. J. Frisch, G. W. Trucks, H. B. Schlegel, G. E. Scuseria, M. A. Robb, J. R. Cheeseman, G. Scalmani, V. Barone, G. A. Petersson, H. Nakatsuji, et al., *Gaussian 16 Revision A.03* (2016), Gaussian Inc. Wallingford CT.

[2] J. Čížek, J. Chem. Phys. **45**, 4256 (1966).

[3] J. P. Perdew, K. Burke, and M. Ernzerhof, Phys. Rev. Lett. **77**, 3865 (1996).

[4] J. P. Perdew, K. Burke, and M. Ernzerhof, Phys. Rev. Lett. **78**, 1396 (1997).

[5] S. H. Vosko, L. Wilk, and M. Nusair, Can. J. Phys. **58**, 1200 (1980).

[6] J. P. Perdew and Y. Wang, Phys. Rev. B **45**, 13244 (1992).

[7] S. Grimme, J. Comput. Chem. **27**, 1787 (2006).

[8] A. D. Becke, J. Chem. Phys. **107**, 8554 (1997).

[9] S. Grimme, J. Chem. Phys. **124**, 034108 (2006).

[10] H. S. Yu, X. He, and D. G. Truhlar, J. Chem. Theory Comput. **12**, 1280 (2016).

[11] H. S. Yu, X. He, S. L. Li, and D. G. Truhlar, Chem. Sci. **7**, 5032 (2016).

[12] J. Chai and M. Head-Gordon, Phys. Chem. Chem. Phys. **10**, 6615 (2008).

[13] T. H. Dunning Jr., J. Chem. Phys. **90**, 1007 (1989).

[14] R. A. Kendall, T. H. Dunning Jr., and R. J. Harrison, J. Chem. Phys. **96**, 6796 (1992).

[15] D. E. Woon and T. H. Dunning Jr., J. Chem. Phys. **98**, 1358 (1993).

[16] K. A. Peterson, D. E. Woon, and T. H. Dunning Jr., J. Chem. Phys. **100**, 7410 (1994).

[17] E. R. Davidson, Chem. Phys. Lett. **260**, 514 (1996).

[18] F. Neese, Wiley Interdisciplinary Reviews: Computational Molecular Science **2**, 73 (2012).

[19] F. Neese, Wiley Interdisciplinary Reviews: Computational Molecular Science **8**, e1327 (2017).

[20] A. Najibi and L. Goerigk, Journal of Chemical Theory and Computation **14**, 5725 (2018).

[21] N. Mardirossian and M. Head-Gordon, Physical Chemistry Chemical Physics **16**, 9904 (2014).

[22] N. Mardirossian and M. Head-Gordon, The Journal of Chemical Physics **144**, 214110 (2016).

[23] S. Grimme, S. Ehrlich, and L. Goerigk, Journal of Computational Chemistry **32**, 1456 (2011).

[24] F. Neese, F. Wennmohs, A. Hansen, and U. Becker, Chemical Physics **356**, 98 (2009).

[25] B. Jeziorski, R. Moszynski, and K. Szalewicz, Chemical Reviews **94**, 1887 (1994).

[26] A. Heßelmann and T. Korona, J. Chem. Phys. **141**, 094107 (2014), ISSN 0021-9606.

[27] H.-J. Werner, P. J. Knowles, G. Knizia, F. R. Manby, and M. Schütz, WIREs Comput Mol Sci **2**, 242 (2012).

[28] H.-J. Werner, P. J. Knowles, G. Knizia, F. R. Manby, M. Schütz, P. Celani, W. Györffy, D. Kats, T. Korona, R. Lindh, et al., *Molpro, version 2018.2, a package of ab initio programs*, https://www.molpro.net (2018), accessed December 2, 2021.

[29] H.-J. Werner, P. J. Knowles, F. R. Manby, J. A. Black, K. Doll, A. Hesselmann, D. Kats, A. Kuehn, T. Korona, D. A. Kreplin, et al., The Journal of Chemical Physics **152**, 144107 (2020).

[30] A. Heßelmann and G. Jansen, Chemical Physics Letters **367**, 778 (2003).

[31] A. Tkatchenko and M. Scheffler, Phys. Rev. Lett. **102**, 073005 (2009).

[32] A. D. Becke and E. R. Johnson, J. Chem. Phys. **127**, 154108 (2007).

[33] A. O. de la Roza and E. R. Johnson, J. Chem. Phys. **136**, 174109 (2012).

[34] M. Dion, H. Rydberg, E. Schröder, D. C. Langreth, and B. I. Lundqvist, Phys. Rev. Lett. **92**, 246401 (2004).

[35] J. Klimeš, D. R. Bowler, and A. Michaelides, J. Phys.: Condens. Matter **22**, 022201 (2010).

[36] J. Klimeš, D. R. Bowler, and A. Michaelides, Phys. Rev. B **83**, 195131 (2011).

[37] K. Berland and P. Hyldgaard, Phys. Rev. B **89**, 035412 (2014).

[38] V. R. Cooper, Phys. Rev. B **81**, 161104(R) (2010).

[39] K. Lee, E. D. Murray, L. Kong, B. I. Lundqvist, and D. C. Langreth, Phys. Rev. B **82**, 081101(R) (2010).

[40] I. Hamada and M. Otani, Phys. Rev. B **82**, 153412 (2010).

[41] I. Hamada, Phys. Rev. B **89**, 121103(R) (2014).

[42] R. Sabatini, T. Gorni, and S. de Gironcoli, Phys. Rev. B **87**, 041108(R) (2013).

[43] S. Grimme, J. Antony, S. Ehrlich, and H. Krieg, J. Chem. Phys. **132**, 154104 (2010).

[44] T. Thonhauser, V. R. Cooper, S. Li, A. Puzder, P. Hyldgaard, and D. C. Langreth, Phys. Rev.

B **76**, 125112 (2007).

[45] G. Román-Pérez and J. M. Soler, Phys. Rev. Lett. **103**, 096102 (2009).

[46] M. Troullier and J. L. Martins, Phys. Rev. B **43**, 1993 (1991).

[47] G. Kresse and J. Joubert, Phys. Rev. B **59**, 1758 (1999).

[48] L. E. Sutton and H. J. M. Bowen, *Tables of Interatomic Distances and Configuration in Molecules and Ions* (The Chemical Society. Special Publication, no. 11, London, 1958).

[49] K. P. Huber and G. Herzberg, *Molecular Spectra and Molecular Structure. IV. Constants of Diatomic Molecules* (Van Nostrand Reinhold, New York, 1979).

[50] J. Plíva, J. W. C. Johns, and L. Goodman, J. Mol. Spectrosc. **148**, 427 (1991).

[51] R. W. G. Wyckoff, *Crystal Structures, Vol. 1* (Interscience, New York, 1963).
